# Unravelling the Role of Post-Junctional M2 Muscarinic Receptors in Cholinergic Nerve-Mediated Contractions of Airway Smooth Muscle

**DOI:** 10.3390/ijms26125455

**Published:** 2025-06-06

**Authors:** Srijit Ghosh, Tuleen Alkawadri, Mark A. Hollywood, Keith D. Thornbury, Gerard P. Sergeant

**Affiliations:** Smooth Muscle Research Centre, Dundalk Institute of Technology, Dublin Road, A91 K584 Dundalk, County Louth, Ireland; srijit.ghosh@dkit.ie (S.G.); tuleen.alkawadri@dkit.ie (T.A.); mark.hollywood@dkit.ie (M.A.H.); keith.thornbury@dkit.ie (K.D.T.)

**Keywords:** muscarinic, airways, smooth muscle, contraction, cholinergic, COPD

## Abstract

It has long been recognised that airway smooth muscle cells (ASMCs) possess an abundance of M2 muscarinic receptors (M2Rs). However, the contribution of postjunctional M2Rs to contractions of airway smooth muscle (ASM) induced by the release of acetylcholine (ACh) from parasympathetic nerves was thought to be minimal. Instead, it was believed that these responses were exclusively mediated by activation of M3Rs. However, evidence is emerging that postjunctional M2Rs may have a greater role than previously realised. In this review, we discuss ACh signalling in airways, highlighting the well-established autoinhibitory role of prejunctional M2Rs and the putative roles of postjunctional M2Rs to cholinergic contractions of ASM. The cellular mechanisms that underpin M2R-dependent contractions of ASM are reviewed, with a particular emphasis on the role of ion channels in these responses. The regulation of M2R signalling pathways by β-adrenoceptor activation is also considered, along with the potential involvement of postjunctional M2Rs in airway diseases such as asthma and chronic obstructive pulmonary disease (COPD).

## 1. Introduction

Airway smooth muscle (ASM) contraction is regulated by the release of acetylcholine (ACh) from parasympathetic nerves [1,2,3,4], which represents the primary bronchoconstrictor neural pathway in the airways [5]. Parasympathetic activity is enhanced in asthma and COPD [6,7,8,9] and is regarded as the major reversible component of airway obstruction [4,10]. Asthmatic patients are hypersensitive to cholinergic agonists [11] and anticholinergics are used to treat both COPD and asthma [12,13,14,15]. ACh induces bronchoconstriction by binding to muscarinic ACh receptors (MRs) on ASM cells (ASMCs) but also targets prejunctional MRs on cholinergic nerve terminals to reduce ACh output, providing a form of autoinhibitory feedback [16,17,18]. MRs can be categorised into five subtypes (MR1–MR5), each encoded by a distinct gene [19], although ASMC mainly possess M2Rs and M3Rs [1,2,3]. The ratio of M2 to M3Rs in ASM is approximately 4:1 in most species [20], yet ACh-induced contractions of ASM are thought to be primarily mediated by M3Rs [21,22]. M3Rs are coupled to G_q_ proteins which induce contractions of ASM by activating the phospholipase C (PLC) signalling cascade to increase inositol trisphosphate (IP_3_) levels and stimulate Ca^2+^ release from intracellular stores [21,23,24]. Despite the abundance of M2Rs in ASM, it is widely thought that they have no direct role in ASM contractions induced by the activation of cholinergic nerves [8,25,26,27]. In contrast, the autoinhibitory function of M2Rs on prejunctional cholinergic nerve terminals in the airways is well established [17,28,29,30,31,32,33,34,35]. These receptors are dysfunctional in patients with asthma [36,37] and in animal models of airway hyperresponsiveness, including antigen challenge [38], virus infection [39], and exposure to ozone [40]. However, evidence is beginning to emerge that postjunctional M2Rs in ASMCs make a greater contribution to the cholinergic contractions of ASM than previously realised. The purpose of this review is to re-examine the role of post junctional M2Rs in ACh-mediated contractions of ASM.

## 2. Role of Postjunctional M2Rs in the Contraction of ASM

M2Rs couple to G_i/o_ proteins that inhibit adenylate cyclase (AC) and reduce cAMP levels [41,42,43]. The G_i/o_ protein family contains a range of α-subunits including G_αi1_–G_αi3_ [44]. All members of the G_i/o_ family contain a cysteine residue close to the C-terminus of their α-subunit which becomes covalently attached to an ADP-ribose moiety in pertussis toxin (PTX) that prevents receptor activation of the G_i/o_ proteins [45]. Therefore, PTX is recognised as a valuable tool for the investigation of G_i/o_ signalling pathways, and the sensitivity of cholinergic contractions to PTX may infer involvement of M2Rs. Kume et al. (1995) [46] and Hirschmann et al. (1999) [47] showed that PTX reduced the amplitude of contractions of ASM evoked by cholinergic agonists, consistent with a role for M2Rs in these responses. Unno et al. (2005) [48] noted that PTX could inhibit contractions of intestinal smooth muscle by the cholinergic agonist carbachol (CCh), but only when low concentrations of the agonist were employed (70% reduction at 0.1 μM and no reduction at 10 μM CCh). This indicated that M2R/G_i/o_ signalling was only involved in responses evoked by relatively low concentrations of ACh, whereas M3R/G_q11_ signalling prevailed at higher agonist concentrations. Semenov et al. (2011) [49] reported that the M2R antagonist AFDX-116 inhibited contractions of murine trachea induced by sub-micromolar concentrations of CCh, but not those evoked by higher concentrations, perhaps suggesting that a similar pattern exists in the airways. Further, indirect evidence in support of a role for M2Rs in cholinergic contractions of ASM came from studies [50,51] which demonstrated that muscarinic agonists evoked robust contractions of ASM taken from M3R knock-out (KO) mice. These responses were not due to compensatory upregulation of M2Rs as the transcriptional expression of M2Rs was not elevated in whole lung preparations or tracheal muscle in M3R KO mice [51]. More definitive evidence in support of a role for M2Rs in ASM contraction came from Stuckmann et al. (2003) [52] who showed that KO of both M2Rs and M3Rs was required to abolish CCh-evoked contractions of tracheal smooth muscle.

**Figure 1:**
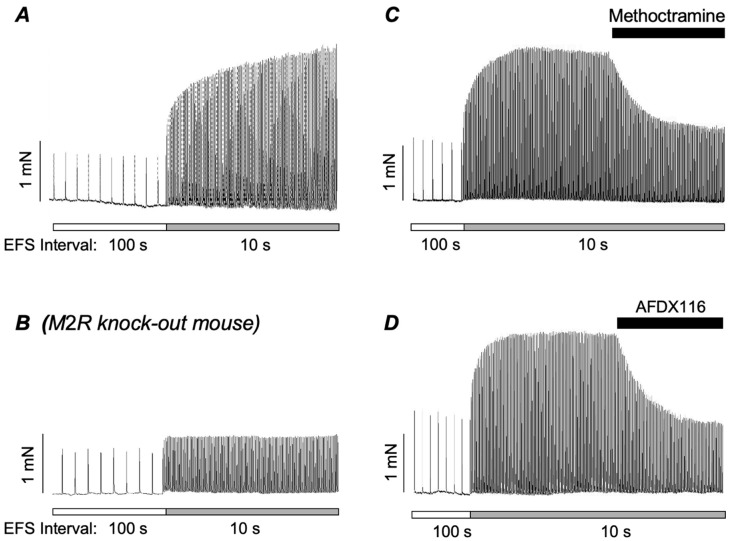
(**A**) Representative trace showing contractions of murine bronchial rings evoked by EFS at 100 and 10 s intervals. Reducing the stimulus interval to 10 s enhanced contraction amplitude. (**B**) Representative trace showing the effect of reducing the stimulus interval from 100 to 10 s on a bronchial ring taken from a M2R KO mouse. (**C**,**D**) Effect of the M2R antagonists, methoctramine (**C**) and AFDX116 (**D**) on contractions of bronchial rings from wild-type mice evoked at 10 s intervals. Adapted from Alkawadri et al. (2021) [53].

A recent study from our laboratory [53] further examined the contribution of postjunctional M2Rs to cholinergic contractions of ASM by investigating whether their involvement was related to the stimulus parameters used to evoke the contractions. It was found that responses to EFS at 2 Hz were affected by the stimulus interval: reduction in the stimulus interval from 100 to 10 s greatly augmented the amplitude of contractions (Figure 1A). This effect was absent in ASM taken from M2R KO mice (Figure 1B) and was reversed by application of the M2R antagonists’ methoctramine and AFDX-116 (Figure 1C,D), demonstrating unequivocally that it was mediated by M2Rs. It should also be noted that, although the augmented responses to 2 Hz EFS at 10 s intervals were mediated by activation of M2Rs, the entire response was abolished by a blockade of M3Rs. This shows that M3R activation was still a prerequisite for these neurogenic contractions and suggests that activation of postjunctional M2Rs sensitises the M3R response. In contrast to the responses to 2 Hz stimulation, contractions evoked by EFS at 20 Hz were unaffected by a reduction in the stimulus interval from 100 to 10 s. Also, unlike the case for 2 Hz EFS, where methoctramine blocked a proportion of the response (Figure 1C,D), responses to 20 Hz stimulation were slightly potentiated by methoctramine. This was consistent with the blockade of prejunctional autoinhibitory M2Rs, which suppress the output of ACh from cholinergic nerves.

Overall, these data indicate that cholinergic contractions of ASM rely on activation of M3Rs, but highlight a prominent role for postjunctional M2Rs in responses evoked by low stimulus frequencies and intervals. This accords with previous studies which indicated that involvement of postjunctional M2Rs in cholinergic contractions of ASM is restricted to responses involving low concentrations of ACh [48,49]. These observations highlight an important role for postjunctional M2Rs in cholinergic contractions of ASM induced by low stimulus frequencies and concentrations of ACh. This may explain why the contribution of postjunctional M2Rs has been underestimated in studies that employ higher-frequency stimulation or higher concentrations of cholinergic agonist to evoke contractions.

## 3. Mechanisms Underlying M2R-Dependent Contractions of ASM

ASM contraction results from activation of myosin light chain kinase (MLCK), which is dependent on an elevation of intracellular Ca^2+^ concentration [54]. There is broad agreement that M3R-dependent contractions of ASM rely on Ca^2+^ release from intracellular stores [55,56,57], but the mechanisms responsible for the M2R-dependent responses are less clear. Here we discuss the cellular pathways underlying these responses.

### 3.1. Role of L-Type Ca^2+^ Channels in M2R-Mediated Contractions of ASM

L-type Ca^2+^ channels (LTCCs) are expressed in airway myocytes in all species studied. Yet, unlike vascular myocytes where it is widely accepted that they mediate contraction, there is no consensus as to their role in ASM [58,59,60]. Although there are many studies that advocate an important role for LTCCs in cholinergic contractions of ASM (comprehensively reviewed in Byron et al., 2014 [60]), there are also many that show no or limited contributions. Furthermore, the results from early clinical trials with Ca^2+^ channel blockers (CCBs) for treating asthma were relatively disappointing compared to their therapeutic efficacy in hypertension and angina [58]. M3Rs are coupled, via G_q/11_, to PLC that generates production of IP_3_, which, in turn, causes sarcoplasmic reticulum (SR) Ca^2+^-release. Therefore, it is easy to see how cholinergic contractions of ASM could occur independently of LTCCs. This led some researchers to conclude that pharmaco-mechanical coupling is the predominant mechanism responsible for mediating cholinergic contractions of ASM, and that electromechanical coupling is only of minor importance [58,59,61]. However, it is difficult to reconcile this view with the fact that ASMCs not only possess LTCCs but also have an impressive inventory of plasmalemmal ion channels that are capable of regulating membrane potential. These include large conductance Ca^2+^-activated K^+^ (BK_Ca_) channels [62], voltage-dependent K^+^ channels, particularly Kv7 channels [60,63], Ca^2+^-activated Cl^−^ channels (CaCCs) [64,65,66,67,68,69,70,71], and a variety of TRP channels [59,72,73]. It is puzzling, therefore, that ASMC should possess both LTCCs and a variety of ways to regulate their open probability if they only play a subsidiary role.

One possible explanation for these disparate findings is to take account of the protocols used extensively in pharmacological experiments. A careful reading of the literature reveals that CCBs are much more effective at reducing responses to submaximal, physiological concentrations of cholinergic agonists than responses elicited by maximal, pharmacological concentrations [60,74,75]. Hence, as discussed by Byron et al. (2014), low concentrations of agonist appear to act predominantly via activation of LTCCs, while higher concentrations act predominantly via IP_3_-mediated Ca^2+^ release from the SR [60]. This also fits with the observation that contractions induced by low concentrations of cholinergic agonist can be inhibited by membrane hyperpolarisation, while those evoked by high concentrations are entirely resistant to this intervention [76].

**Figure 2:**
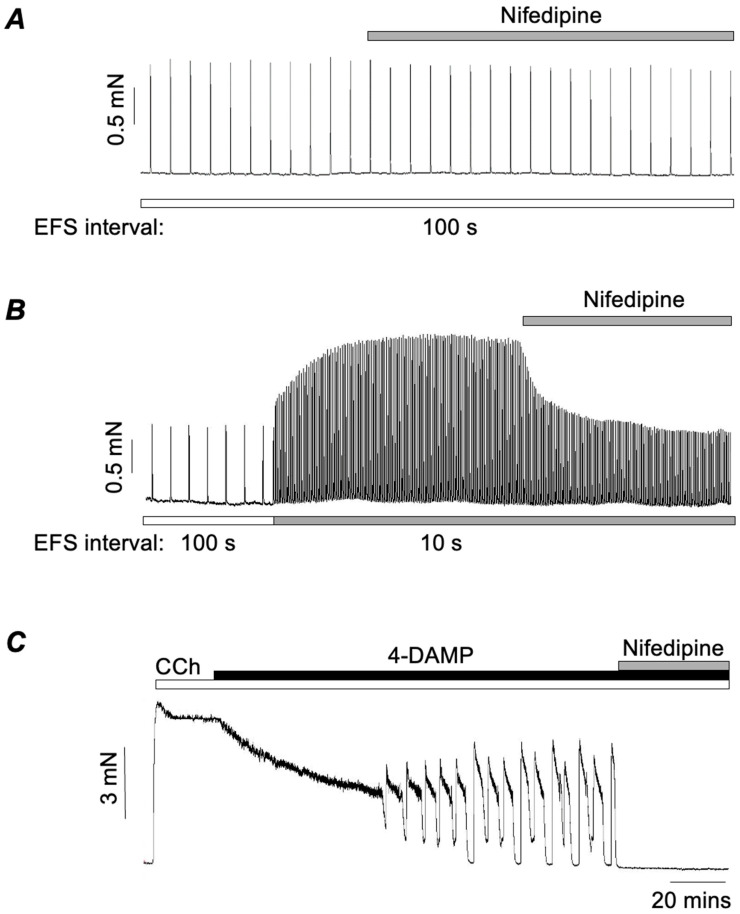
(**A,B**) Representative traces showing the effects of nifedipine on contractions of murine bronchial rings evoked by EFS at 100 s (**A**) and 10 s intervals (**B**). (**C**) Effect of nifedipine on contractions of bronchial rings evoked by the cholinergic agonist carbachol (CCh) in the presence of the M3R antagonist, 4-DAMP. Adapted from Ghosh et al. (2025) [77].

As noted above, M2Rs exert their effects at lower agonist concentrations, leading us to postulate that M2R-mediated responses depend on activation of LTCCs. To test this idea, Ghosh et al. (2025) [77] showed that nifedipine, an LTCC blocker, had no effect on EFS responses evoked at 100 s intervals, indicating that these M3R-mediated responses did not depend on LTCCs (Figure 2A). In stark contrast, when the stimulus interval was switched to 10 s to unmask the augmented responses due to activation of M2Rs, nifedipine completely reversed the augmentation (Figure 2B). Similarly, when the M3R antagonist 4-DAMP (3 nM) was applied to tissues pre-contracted with 300 nM CCh to isolate M2R-mediated phasic contractions, these were also abolished by nifedipine (Figure 2C). In contrast, nifedipine only reduced maximal contractions induced by CCh (10 μM) in the absence of 4-DAMP by around 20–30% (Dwivedi et al., 2023) [75]. Taken together, these results strongly support the idea that M2R-mediated contractions are almost entirely dependent on Ca^2+^ influx through LTCCs, while these channels contribute only a little to M3R-dependent responses.

### 3.2. Role of TMEM16A Ca^2+^-Activated Cl^−^ Channels in M2R-Dependent Contractions of ASM

**Figure 3:**
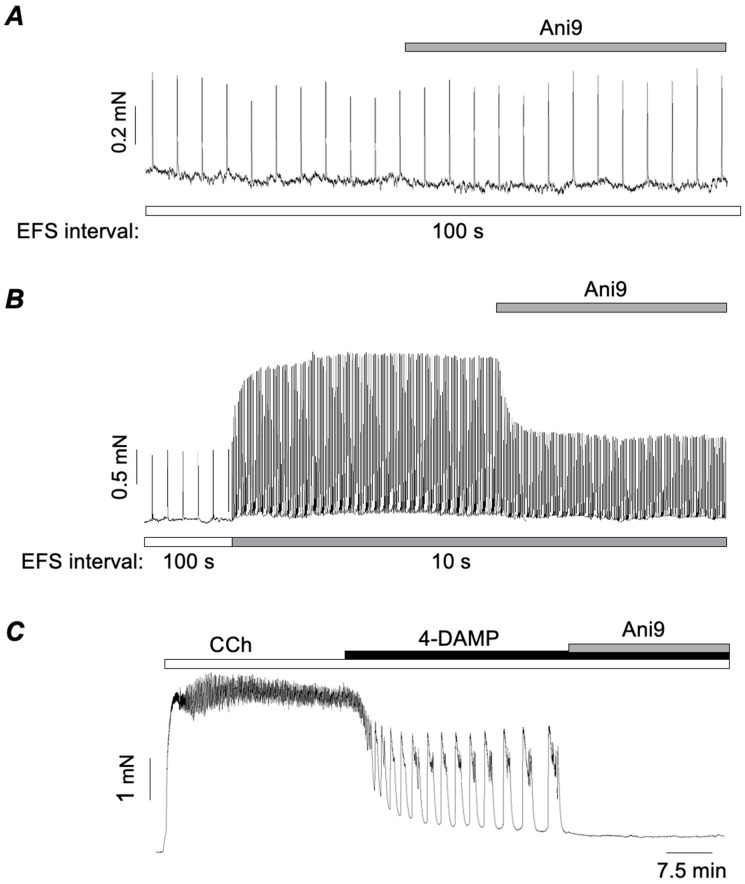
(**A**,**B**) Representative traces showing the effects of Ani9 on contractions of murine bronchial rings evoked by EFS at 100 s (**A**) and 10 s intervals (**B**). (**C**) Effect of Ani9 on contractions of bronchial rings evoked by the cholinergic agonist carbachol (CCh) in the presence of the M3R antagonist 4-DAMP. Adapted from Ghosh et al. (2025) [77].

LTCCs are activated by membrane depolarisation; therefore, their involvement in M2R-mediated responses implies that other ion channels or another electrogenic mechanisms are involved. As alluded to above, an array of ion channels is expressed in ASM. Here we will consider the role of CaCCs, before discussing other alternatives. Many early studies established the presence of CaCCs in ASM [64,65,66,67,68], which are now known to be encoded by TMEM16A [69,70,71]. Wang et al. (2018) [71] showed that genetic KO of TMEM16A greatly attenuated contractile responses to histamine and the thromboxane agonist, U46619. In contrast, responses induced by maximal concentrations of methacholine were unaffected in TMEM16A KO mice. However, responses induced by lower concentrations were 50% smaller than those in wild-type mice. Several studies concluded that TMEM16A channels are important for excitation coupling of responses to cholinergic agonists in ASM, even at higher concentrations [70,78,79]. However, these studies relied, at least in part, on the TMEM16A blocker benzbromarone, the specificity of which has since been questioned [75]. Dwivedi et al. (2023) found that benzbromarone inhibited CCh-induced contractions over the concentration range of 0.1–10 μM [75]. Worryingly, however, it had a markedly greater inhibitory effect than nifedipine, and a further inhibitory effect in tissues that had already been exposed to nifedipine. These data indicated that benzbromarone had one or more off-target effects beyond blocking the membrane depolarisation responsible for activating LTCCs. Dwivedi et al. (2023) also showed that benzbromarone, MONNA, and, to a lesser extent, CaCC_inh_-A01 caused SR Ca^2+^ release in isolated mouse bronchial myocytes, but that Ani9, another TMEM16A blocker, did not [75]. However, Ani9 failed to block CCh-evoked contractions (0.1–10 μM), although it abolished phasic contractions induced by 5-HT. Taken together, this evidence suggested that TMEM16A CaCCs had no role in mediating cholinergic contractions in ASM. However, given that nifedipine inhibited the M2R-mediated contractions (Figure 2B,C), but had little effect on M3R-mediated contractions (Figure 2A), we decided to test if the same was true for Ani9 [77]. Ani9 had no effect on M3R-mediated contractions evoked by EFS at 100 s intervals but abolished the M2R-mediated enhancement of EFS contractions, observed at 10 s stimulus intervals (Figure 3B). Furthermore, Ani9 also abolished M2R-mediated contractions induced by CCh in the presence of 4-DAMP (Figure 3C). Hence, we concluded that TMEM16A CACCs are involved in M2R-mediated contractions of ASM, but not in contractions mediated only by M3Rs.

Since TMEM16A channels are involved in M2R-mediated responses, two questions arise. Firstly, what is the source of Ca^2+^ responsible for their activation, and secondly, what is the sequence of events, following the stimulation of M2Rs, that leads to their activation? At present, there are no clear answers to these questions, but, based on experimental evidence, it is possible to propose some possibilities. An obvious candidate for activating TMEM16A CACCs would be Ca^2+^ released from the SR by IP_3_ following M3R stimulation. However, lacking in this argument is an obvious link to M2Rs. One possible way to link M2Rs to this process would be inhibition of the SERCA pump. In vascular smooth muscle, the superficial SR is closely opposed to the plasma membrane such that it takes up Ca^2+^ as it enters the cell, thus preventing it from reaching the contractile proteins, a phenomenon known as the ‘superficial buffer barrier’ [80,81,82]. Such a phenomenon is well established in some vascular smooth muscles, but it has been less well studied in airway smooth muscle [80,81,82]. Nevertheless, Janssen et al. (1999) [83] showed that the inhibition of SERCA in canine ASM with cyclopiazonic acid potentiated contractures induced by KCl, suggesting that a similar phenomenon may also exist in ASM. SERCA is stimulated by cAMP, which, via protein kinase A (PKA), phosphorylates a small associated protein called phospholamban (PLN). The phosphorylation of PLN causes it to dissociate from SERCA, thus increasing SERCA pump activity [84,85]. As M2Rs are coupled to adenylate cyclase via G_i/o_ proteins, their activation will reduce production of cAMP and hence inhibit the SERCA pump. Thus, it is possible to propose a sequence whereby stimulation of M3Rs causes SR Ca^2+^ release via IP_3_ which, in turn, activates TMEM16A CACCs resulting in membrane depolarisation and Ca^2+^ influx via LTCCs. In the absence of M2R stimulation, most of this Ca^2+^ would be buffered by the SERCA pump and fail to reach the contractile proteins deeper within the cell. However, if M2Rs are simultaneously activated with M3Rs, then inhibition of the SERCA pump would allow the Ca^2+^ to reach its target to produce a partially M2R-dependent contraction. In support of this idea, Ghosh et al. (2025) [77] showed that thapsigargin, a SERCA pump inhibitor, potentiated EFS-induced contractions of ASM evoked at 100 s intervals to a similar extent as that induced by reducing the stimulus interval to 10 s. Thus, SERCA inhibition had similar stimulatory effects on EFS-evoked contractions of ASM as those induced by the activation of M2Rs. Furthermore, the enhancement induced by thapsigargin was completely reversed by either nifedipine or Ani9, similar to the effect of these agents on the M2R-induced enhancement of EFS responses.

While the above hypothesis might explain how M2R activation potentiates EFS responses, it does not hold up for the M2R-mediated phasic contractions evoked by CCh in the presence of 4-DAMP, such as those shown in Figure 2B and Figure 3B. In these experiments M3Rs were blocked, so the initial trigger for the above sequence, namely the IP_3_-mediated SR Ca^2+^ release, would be absent. In this scenario, it is necessary to propose a different cause for depolarisation, other than the activation of TMEM16A CACCs, such as the inhibition of K^+^ channels or the activation of non-specific cation channels. Some of these are discussed below, but meanwhile, if TMEM16A channels do not initiate the depolarisation, how does their blockade lead to the inhibition of the responses? We hypothesise that the activation of M2Rs inhibits the SERCA pump [77]. This reduces Ca^2+^ buffering and allows Ca^2+^ entering via LTCCs to (1) access the contractile proteins and (2) activate TMEM16A CACCs, thus potentiating and sustaining the depolarisation via positive feedback. The advantage of activating TMEM16A is that it clamps the membrane potential at the Cl^−^ equilibrium potential, believed to be around −24 mV in smooth muscle [86]. This also coincides with the potential at which Ca^2+^ influx can be maintained because of the ‘window current’ phenomenon, whereby a proportion of LTCCs always remains open as a result of the balance between the activation and inactivation of LTCCs [87].

### 3.3. Involvement of K^+^ Channels or Non-Specific Cation Channels?

The closure of K^+^ channels or opening of non-specific cation channels has the potential to initiate the depolarisation of ASMC. With regard to K^+^ channels in ASM, the focus has mainly been on BK_Ca_ channels and K_V_7 voltage-dependent K^+^ channels. We will consider these briefly in turn, specifically with regard to their possible involvement in M2R responses. For more detailed reviews of these channels in ASM, the reader is referred to Kume (2014) [62] and Byron et al. (2014) [60].

Methacholine was shown to inhibit single BK_Ca_ channels from ASMC via a PTX-sensitive mechanism, implying M2R-mediated coupling via G_i/o_ proteins [88,89]. Since the open probability of BK_Ca_ channels is increased by phosphorylation by PKA, a reduction in cAMP production by G_i/o_ would be expected to reduce this effect and close BK_Ca_ channels [89]. In addition, a later study by Zhou et al. (2008) [90] used a combination of M2R and BK_Ca_ co-expressed in HEK293 cells to show that the βγ subunits from G_i/o_ proteins could directly inhibit the pore-forming α-subunits of BK_Ca_ channels. The βγ subunits also inhibited BK_Ca_ channels via a second pathway involving the activation of protein kinase C (PKC) secondary to activating the PLCβ2 isoform of PLC. However, although cholinergic agonists were able to inhibit BK_Ca_ via M2Rs, another study suggested that this mechanism plays only a minor role in in mediating the contractions of ASM induced by M2Rs [49].

Several K_V_7 subtypes (K_V_7.1–K_V_7.5) have been detected in human, guinea pig, rat, and mouse ASM [60,91,92]. These channels have the capacity to regulate the resting membrane potential as they activate at relatively negative potentials compared to other K^+^ channels [60]. They are also suppressed by cholinergic agonists and histamine and thus can account, at least in part, for the depolarisation produced by these agonists [91]. There are several mechanisms by which cholinergic agonists could suppress K_V_7 currents. Firstly, all K_V_7 subtypes (K_V_7.1–K_V_7.5) bind to phosphatidylinositol 4,5-bisphosphate (PIP_2_), which facilitates channel opening; hence, PIP_2_ depletion closes the channel [93]. The activation of M3Rs would therefore, via the activation of PLC, be expected to inhibit Kv7 channels with resultant depolarisation. It has also been shown that cAMP, working via both PKA and ‘exchange protein directly activated by cAMP’ (EPAC), increased Kv7 channel open probability [63,94]. Hence, a reduction in cAMP levels via the activation of G_i/o_ could provide a possible link between M2R stimulation and the inhibition of Kv7 channels.

**Figure 4:**
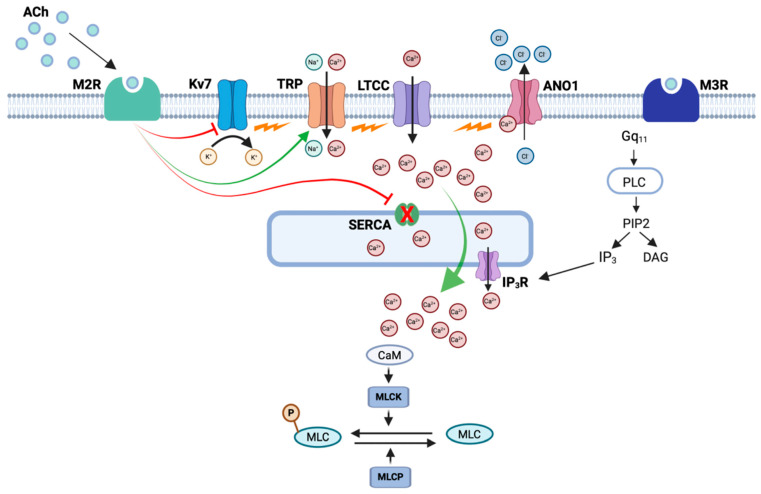
Model showing cellular mechanism underlying contractions of ASM induced by EFS at 100 and 10 s intervals. At 100 s intervals, EFS-evoked contractions of ASM are mediated by the activation of M3Rs leading to the release of Ca^2+^ from the sarcoplasmic reticulum via IP_3_Rs. Ca^2+^ influx via L-type Ca^2+^ channels (LTCCs) is buffered by the activity of SERCA pumps on the peripheral sarcoplasmic reticulum. Reducing the stimulus interval to 10 s leads to the activation of M2Rs, which inhibits SERCA activity, resulting in reduced buffering in Ca^2+^ influx via LTCCs. Increased Ca^2+^ levels in the subsarcolemmal space activates Ano1 Ca^2+^-activated Cl^−^ channels leading to Cl^−^ efflux, membrane depolarization, and further activation of LTCCs. Increased cytosolic Ca^2+^ levels increase contraction amplitude. Created with BioRender.com. Adapted from Ghosh et al. (2025) [77].

ASM cells also possess non-selective cation channels that mediate inward currents (*I*_cat_). These are activated by cholinergic agonists, thus providing another potential depolarising pathway for the activation of LTCCs [65,66,72,73,95]. Interestingly, the M2R antagonist methoctramine reduced the amplitude of *I*_cat_ induced by methacholine, indicating a requirement for M2Rs in the activation of this pathway [66]. Furthermore, methacholine failed to evoke *I*_cat_ in the presence of PTX or antibodies directed towards G_i/o_ proteins, consistent with a role for M2Rs. However, the blockade of M3Rs also abolished *I*_cat_, suggesting that both M3R and M2Rs were involved in the response [66]. Interestingly, M2R stimulation alone could activate *I*_cat_ if intracellular Ca^2+^ was elevated by the simultaneous application of caffeine. This suggests that M3R involvement was mediated by their ability to cause Ca^2+^ release, rather than by a direct action on the channels. Several studies have suggested that the molecular identity of the channels underlying *I*_cat_ in ASM is TRPC3 [65,66,95]. However, we found that Pyr3, a selective TRPC3 inhibitor, had no effect on the M2R-mediated enhancement of EFS responses in murine ASM, suggesting that TRPC3 is not involved in this particular response (unpublished observations). This does not preclude the involvement of other TRPC channels, as most of the members of this family (TRPC1-7) are expressed in ASM [66]. A model detailing how these signalling pathways could interact to elicit contractions in ASM in response to M2R stimulation is provided in Figure 4.

## 4. Modulation of M2R-Dependent Contractions of ASM

ASM possesses β_2_-adrenoceptors (β_2_-ARs) that are coupled to G_s_-proteins, which activate adenylate cyclase to elevate cytosolic cAMP levels and stimulate protein kinase A (PKA) [10,96]. β-AR agonists are potent bronchodilators and are used to prevent, or alleviate, the symptoms of obstructive lung conditions such as COPD and asthma [10,97]. The stimulation of M2Rs is thought to counteract the inhibitory effects of β-AR activation on ASM contraction by activating G_i/o_-proteins that decrease adenylate cyclase activity and reduce cAMP levels [42,98,99,100,101,102]. Therefore, it is thought that M2Rs provide a functional antagonism to the inhibitory effects of β-AR activation on ASM, and many studies have shown that M2R antagonists potentiate the inhibitory effects of β-AR on ASM contractility.

β-AR agonists attenuate ACh-induced contractions of ASM [103,104]. Since the contractile effects of ACh were primarily thought to be mediated by activation of M3Rs, it was believed that the inhibitory effects of β2-AR agonists resulted from the inhibition of M3R-dependent signalling pathways in ASMCs [97,105]. However, as a greater role for postjunctional M2Rs in cholinergic contractions emerged, it raised the possibility that these responses could also be modulated by β-AR agonists. Alkawadri et al. (2022) [106] demonstrated that M2R-dependent contractions of murine ASM were abolished by the activation of β-ARs. They showed that the β-AR agonist denopamine (1) abolished 4-DAMP-resistant contractions of ASM that were induced by CCh; (2) inhibited the M2R-dependent enhancement of EFS-evoked contractions brought about a reduction in the stimulus interval; and (3) was more efficacious in inhibiting contractions of ASM evoked by EFS at 2 Hz (which involve M2Rs) compared to those at 20 Hz which do not. Therefore, these findings suggested that the bronchodilator effects of β-AR agonists may involve the inhibition of M2R-dependent signalling pathways in ASMCs.

## 5. A Role for M2Rs in Asthma and COPD?

Asthma and COPD are associated with increased activity of parasympathetic cholinergic nerves and increased hyperresponsiveness to ACh agonists [4,8,9,11]. However, despite the upregulation of cholinergic activity in asthma, this does not appear to be associated with a change in M3R functionality. For example, Whicker et al. (1990) [107] showed that antigen challenge in guinea pig ASM led to increased sensitivity to CCh, but this was not associated with a change in the binding of a radiolabelled muscarinic receptor ligand. Similarly, Haddad et al. (1996) [108] showed that there was no significant difference in the affinity or the density of muscarinic receptors in peripheral lung samples taken from asthmatics compared with non-asthmatics. In addition, single nucleotide polymorphisms in the promoter region of the human M3R gene (*CHRM3*) did not appear to be associated with asthma as there was no difference in the recorded frequency of SNPs among asthmatic patients and healthy control subjects (Donfack et al., 2003) [109].

It is well established that asthma and COPD are associated with dysfunction in prejunctional M2Rs; however, there is also some indication that postjunctional M2Rs may also play a role in the pathogenesis of these conditions. The primary lines of evidence in this regard are the sensitivity of heightened cholinergic responses of ASM to PTX and the increased expression of G_i/0_ proteins that couple to M2Rs in experimental models of asthma. For example, Hakonarson et al. (1995) [110] showed that rabbit tracheal smooth muscle that was passively sensitized with serum from atopic asthmatics had enhanced contractile responses to ACh that were attenuated by PTX. Furthermore, G_i_α subunit expression was increased in tissues treated with serum from atopic asthmatics. Similarly, Chiba et al. (2001) [111] demonstrated that augmented responses to ACh in antigen-treated ASM of rats were reduced by PTX and that G_i_α_3_ protein levels were greatly enhanced. The inflammatory mediators, IL-1β and TNF-α, play important roles in the pathogenesis of the airway inflammatory response in asthma [112,113,114,115,116]. Hakonarson et al. (1996) [117] showed that IL-1β and, to a lesser extent, TNF-α attenuated β-AR–mediated relaxations of ASM that were precontracted with ACh. It was suggested that the effects of IL-1β were due to activation of an M2R-signalling pathway as methoctramine potentiated isoproterenol-induced relaxations of tissues treated with IL-1β, but not controls. This study also found that the expression of G_i_α_2_ and G_i_α_3_ subunits was enhanced in ASM treated with IL-1β. Therefore, it is possible that postjunctional M2Rs may not be directly upregulated in airway disease but that M2R-dependent signalling pathways could be enhanced as a result of increased G_i_α subunit expression and enhanced G_i_α/M2R coupling.

**Figure 5:**
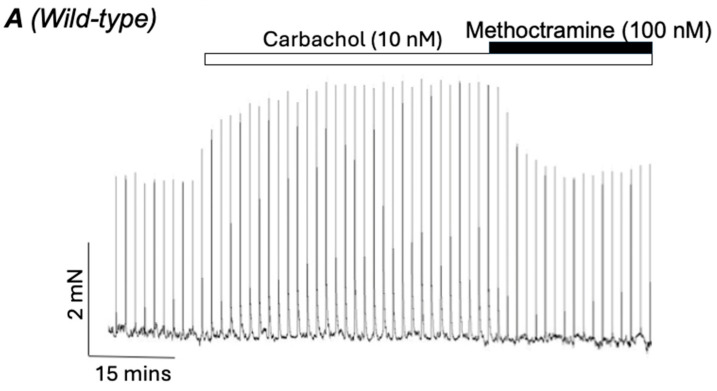

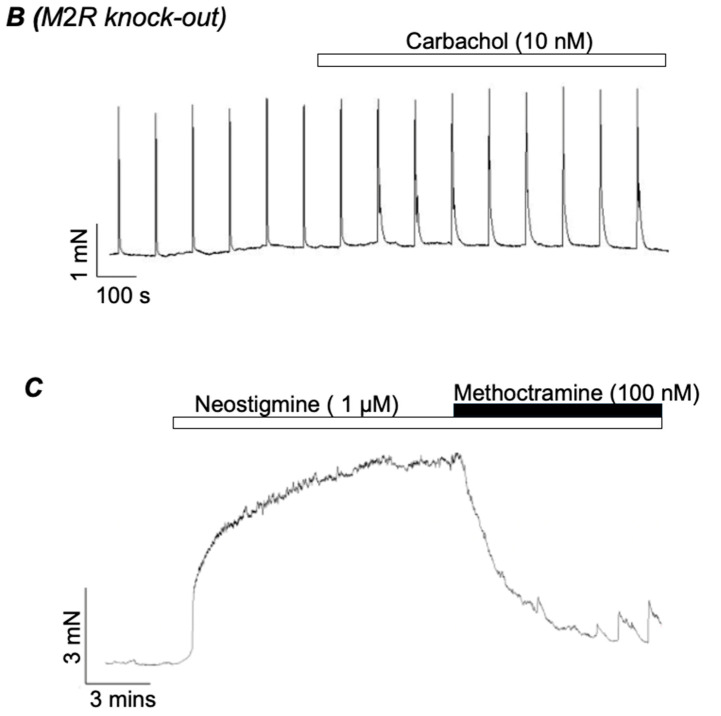
Representative trace showing that CCh (10 nM) enhanced the amplitude of EFS-evoked contractions of ASM (2 Hz, 100 s intervals) and that this effect was reversed by methoctramine (**A**). (**B**) is a representative trace showing that CCh (10 nM) did not affect EFS-evoked contractions of ASM taken from M2R KO mice. (**C**) is a representative trace showing that methoctramine (100 nM) inhibited contractions of ASM induced by the acetylcholinesterase inhibitor neostigmine (1 µM). Adapted from Alkawadri et al. (2021) [53].

Huang et al. (2024) [118] recently reported that elements of the non-neuronal cholinergic system, including MRs, and ACh-related enzymes, were upregulated in the airways of patients with severe asthma. Interestingly, Alkawadri et al. (2021) [53] showed that application of a subthreshold concentration of carbachol (10 nM) increased the amplitude of EFS-evoked contractions of ASM and that this effect was reversed by methoctramine (Figure 5A) and was absent in M2R KO mice (Figure 5B). They also showed that contractions of ASM, induced by the acetylcholinesterase inhibitor neostigmine, were inhibited by methoctramine *(*Figure 5C). These observations led the authors to postulate that postjunctional M2Rs were involved in ASM contractions induced by a small increase in ambient ACh concentration, such as that arising from non-neuronal sources [53]. These observations suggest, then, that postjunctional M2Rs could be targeted for the treatment of asthma. However, M2R antagonists could also inhibit prejunctional autoinhibitory M2Rs, which would enhance neuronal ACh output. Therefore, from a therapeutic point of view, there is currently a preference to use long-acting muscarinic antagonists (LAMAs) that dissociate more slowly from M3Rs compared to M2Rs and therefore provide a kinetic selectivity for M3 over M2Rs [119]. To selectively target responses evoked by activation of postjunctional M2Rs requires a better understanding of the cellular pathways responsible for these effects. Therefore, future studies should focus on elucidating the mechanisms underlying M2R-dependent contractions of ASM and investigate if these pathways are altered in asthma or COPD.

## 6. Conclusions

There is now accumulating evidence to suggest that postjunctional M2Rs make an important contribution to cholinergic contractions of ASM, especially to responses evoked by low concentrations of ACh and low-frequency EFS. Cholinergic nerve-evoked contractions of ASM are reliant on M3Rs, but stimulation of postjunctional M2Rs exerts a profound enhancement in these responses. We speculate that M3Rs located at nerve-smooth muscle junctions represent the primary target for neuronally released ACh, while M2Rs are located extrajunctionally and may be activated by the overspill of junctional ACh, or by ACh from non-neuronal sources which may occur in pathophysiological situations. To determine if postjunctional M2Rs could be targeted for the treatment of airway disease, more work is required to elucidate the processes leading to activation of postjunctional M2Rs and the cellular mechanisms responsible for their effects.

## References

[1] Zaagsma J., Roffel A.F., Meurs H. (1997). Muscarinic control of airway function. Life Sci..

[2] Fryer A.D., Jacoby D.B. (1998). Muscarinic receptors and control of airway smooth muscle. Am. J. Respir. Crit. Care Med..

[3] Barnes P.J. (2004). The role of anticholinergics in chronic obstructive pulmonary disease. Am. J. Med. Suppl..

[4] Cazzola M., Page C.P., Calzetta L., Matera M.G. (2012). Pharmacology and therapeutics of bronchodilators. Pharmacol. Rev..

[5] Gosens R., Gross N. (2018). The mode of action of anticholinergics in asthma. Eur. Respir J..

[6] Gross N.J., Co E., Skorodin M.S. (1989). Cholinergic bronchomotor tone in COPD. Estimates of its amount in comparison with that in normal subjects. Chest.

[7] Gross N.J., Skorodin M.S. (1984). Role of the parasympathetic system in airway obstruction due to emphysema. N. Engl. J. Med..

[8] Coulson F.R., Fryer A.D. (2003). Muscarinic acetylcholine receptors and airway diseases. Pharmacol. Ther..

[9] Gosens R., Zaagsma J., Meurs H., Halayko A.J. (2006). Muscarinic receptor signaling in the pathophysiology of asthma and COPD. Respir. Res..

[10] Matera M.G., Page C.P., Calzetta L., Rogliani P., Cazzola M. (2020). Pharmacology and Therapeutics of Bronchodilators Revisited. Pharmacol. Rev..

[11] Jacoby D.B., Fryer A.D. (2001). Anticholinergic therapy for airway diseases. Life Sci..

[12] Cazzola M., Rogliani P., Matera M.G. (2020). The latest on the role of LAMAs in asthma. J. Allergy Clin. Immunol..

[13] Cazzola M., Luigino Calzetta L., Matera M.G. (2021). Long-acting muscarinic antagonists and small airways in asthma: Which link?. Allergy.

[14] Cazzola M., Rogliani P., Matera M.G. (2023). Might It Be Appropriate to Anticipate the Use of Long-Acting Muscarinic Antagonists in Asthma?. Drugs.

[15] Agache I., Adcock I.M., Akdis C.A., Akdis M., Bentabol-Ramos G., van den Berge M., Boccabella C., Canonica W.G., Caruso C., Couto M. (2025). The Bronchodilator and Anti-Inflammatory Effect of Long-Acting Muscarinic Antagonists in Asthma: An EAACI Position Paper. Allergy.

[16] Fryer A.D., Maclagan J. (1984). Muscarinic inhibitory receptors in pulmonary parasympathetic nerves in the guinea-pig. Br. J. Pharmacol..

[17] Blaber L.C., Fryer A.D., Maclagan J. (1985). Neuronal muscarinic receptors attenuate vagally-induced contraction of feline bronchial smooth muscle. Br. J. Pharmacol..

[18] Faulkner D., Fryer A.D., Maclagan J. (1986). Postganglionic muscarinic inhibitory receptors in pulmonary parasympathetic nerves in the guinea-pig. Br. J. Pharmacol..

[19] Caulfield M.P., Birdsall N.J. (1998). International Union of Pharmacology. XVII. Classification of muscarinic acetylcholine receptors. Pharmacol. Rev..

[20] Belmonte K.E. (2005). Cholinergic pathways in the lungs and anticholinergic therapy for chronic obstructive pulmonary disease. Proc. Am. Thorac. Soc..

[21] Roffel A., Meurs H., Elzinga C., Zaagsma J. (1990). Characterization of the muscarinic receptor subtype involved in phosphoinositide metabolism in bovine tracheal smooth muscle. Br. J. Pharmacol..

[22] Fisher J.T., Vincent S.G., Gomeza J., Yamada M., Wess J. (2004). Loss of Vagally Mediated Bradycardia and Bronchoconstriction in Mice Lacking M_2_ or M_3_ Muscarinic Acetylcholine Receptors. FASEB J..

[23] Chilvers E.R., Batty I.H., Barnes P.J., Nahorski S.R. (1990). Formation of inositol polyphosphates in airway smooth muscle after muscarinic receptor stimulation. J. Pharmacol. Exp. Ther..

[24] Grandordy B.M., Cuss F.M., Sampson A.S., Palmer J.B., Barnes P.J. (1986). Phosphatidylinositol response to cholinergic agonists in airway smooth muscle: Relationship to contraction and muscarinic receptor occupancy. J. Pharmacol. Exp. Ther..

[25] Watson N., Magnussen H., Rabe K.F. (1995). Pharmacological characterization of the muscarinic receptor subtype mediating contraction of human peripheral airways. J. Pharmacol. Exp. Ther..

[26] Barnes P.J. (1993). Muscarinic receptor subtypes in the airways. Life Sci..

[27] Roux E., Molimard M., Savineau J.P., Marthan R. (1998). Muscarinic stimulation of airway smooth muscle cells. Gen. Pharmacol. Vasc. Syst..

[28] Baker D.G., Don H.F., Brown J.K. (1992). Direct measurement of acetylcholine release in guinea pig trachea. Am. J. Physiol.-Lung Cell. Mol. Physiol..

[29] Aas P., Maclagan J. (1990). Evidence for prejunctional M_2_ muscarinic receptors in pulmonary cholinergic nerves in the rat. Br. J. Pharmacol..

[30] Eltze M., Galvan M. (1994). Involvement of muscarinic M_2_ and M_3_, but not of M_1_ and M_4_ receptors in vagally stimulated contractions of rabbit bronchus/trachea. Pulm. Pharmacol..

[31] Ito Y., Yoshitomi T. (1988). Autoregulation of acetylcholine release from vagus nerve terminals through activation of muscarinic receptors in the dog trachea. Br. J. Pharmacol..

[32] Minette P.A., Barnes P.J. (1988). Prejunctional inhibitory muscarinic receptors on cholinergic nerves in human and guinea pig airways. J. Appl. Physiol..

[33] Patel H.J., Barnes P.J., Takahashi T., Tadjkarimi S., Yacoub M.H., Belvisi M.G. (1995). Evidence for prejunctional muscarinic autoreceptors in human and guinea pig trachea. Am. J. Respir. Crit. Care Med..

[34] Berge R.E.T., Zaagsma J., Roffel A.F. (1996). Muscarinic inhibitory autoreceptors in different generations of human airways. Am. J. Respir. Crit. Care Med..

[35] Wang Z.W., Yu M.F., Robinson N.E., Derksen F.J. (1995). Acetylcholine re- lease from airway cholinergic nerves in horses with heaves, an airway obstructive disease. Am. J. Respir. Crit. Care Med..

[36] Minette P.A., Lammers J.W., Dixon C.M., McCusker M.T., Barnes P.J. (1989). A muscarinic agonist inhibits reflex bronchoconstriction in normal but not in asthmatic subjects. J. Appl. Physiol..

[37] Ayala L.E., Ahmed T. (1989). Is there loss of protective muscarinic receptor mechanism in asthma?. Chest.

[38] Fryer A.D., Wills-Karp M.J. (1991). Dysfunction of M2-muscarinic receptors in pulmonary parasympathetic nerves after antigen challenge. J. Appl. Physiol..

[39] Fryer A.D., Jacoby D.B. (1991). Parainfluenza virus infection damages inhibitory M_2_ muscarinic receptors on pulmonary parasympathetic nerves in the guinea-pig. Br. J. Pharmacol..

[40] Schultheis A.H., Bassett D.J., Fryer A.D. (1994). Ozone-induced airway hyperresponsiveness and loss of neuronal M2 muscarinic receptor function. J. Appl. Physiol..

[41] Eglen R.M., Reddy H., Watson N., Challiss R. (1994). Muscarinic acetylcholine receptor subtypes in smooth muscle. Trends Pharmacol. Sci..

[42] Ehlert F.J., Ostrom R.S., Sawyer G.W. (1997). Subtypes of the muscarinic receptor in smooth muscle. Life Sci..

[43] Ehlert F.J. (2003). Contractile role of M_2_ and M_3_ muscarinic receptors in gastrointestinal, airway and urinary bladder smooth muscle. Life Sci..

[44] Wettschureck N., Offermanns S. (2005). Mammalian G proteins and their cell type specific functions. Physiol. Rev..

[45] Locht C., Coutte L., Mielcarek N. (2011). The ins and outs of pertussis toxin. FEBS J..

[46] Kume H., Mikawa K., Takagi K., Kotlikoff M.I. (1995). Role of G proteins and KCa channels in the muscarinic and beta-adrenergic regulation of airway smooth muscle. Am. J. Physiol.-Lung Cell. Mol. Physiol..

[47] Hirshman C.A., Lande B., Croxton T.L. (1999). Role of M_2_ muscarinic receptors in airway smooth muscle contraction. Life Sci..

[48] Unno T., Matsuyama H., Sakamoto T., Uchiyama M., Izumi Y., Okamoto H., Yamada M., Wess J., Komori S. (2005). M(2) and M(3) muscarinic receptor-mediated contractions in longitudinal smooth muscle of the ileum studied with receptor knockout mice. Br. J. Pharmacol..

[49] Semenov I., Wang B., Herlihy J.T., Brenner R. (2011). BK channel β1 subunits regulate airway contraction secondary to M2 muscarinic acetylcholine receptor mediated depolarization. J. Physiol..

[50] Stengel P.W., Yamada M., Wess J., Cohen M.L. (2002). M(3)-receptor knockout mice: Muscarinic receptor function in atria, stomach fundus, urinary bladder, and trachea. Am. J. Physiol. Regul. Integr. Comp. Physiol..

[51] Schlenz H., Kummer W., Jositsch G., Wess J., Krasteva G., Shaik F.A., Medapati M.R., Chelikani P., Mahavadi S., Bhattacharya S. (2010). Muscarinic receptor-mediated bronchoconstriction is coupled to caveolae in murine airways. Am. J. Physiol.-Lung Cell Mol. Physiol..

[52] Struckmann N., Schwering S., Wiegand S., Gschnell A., Yamada M., Kummer W., Wess J., Haberberger R.V. (2003). Role of muscarinic receptor subtypes in the constriction of peripheral airways: Studies on receptor-deficient mice. Mol. Pharmacol..

[53] Alkawadri T., McGarvey L.P., Mullins N.D., Hollywood M.A., Thornbury K.D., Sergeant G.P. (2021). Contribution of Postjunctional M2 Muscarinic Receptors to Cholinergic Nerve-Mediated Contractions of Murine Airway Smooth Muscle. Function.

[54] Zhang W.-C., Peng Y.-J., Zhang G.-S., He W.-Q., Qiao Y.-N., Dong Y.-Y., Gao Y.-Q., Chen C., Zhang C.-H., Li W. (2010). Myosin light chain kinase is necessary for tonic airway smooth muscle contraction. J. Biol. Chem..

[55] Barnes P.J. (1990). Muscarinic receptors in airways: Recent developments. J. Appl. Physiol.

[56] Roux E., Guibert C., Savineau J., Marthan R. (1997). [Ca^2+^](i) oscillations induced by muscarinic stimulation in airway smooth muscle cells: Receptor subtypes and correlation with the mechanical activity. Br. J. Pharmacol..

[57] Liu Q.-H., Zheng Y.-M., Wang Y.-X. (2007). Two distinct signaling pathways for regulation of spontaneous local Ca^2+^ release by phospholipase C in airway smooth muscle cells. Pflug. Arch. Eur. J. Physiol..

[58] Janssen L. (2002). Ionic mechanisms and Ca(^2+^) regulation in airway smooth muscle contraction: Do the data contradict dogma?. Am. J. Physiol.-Lung Cell. Mol. Physiol..

[59] Perez-Zoghbi J.F., Karner C., Ito S., Shepherd M., Alrashdan Y., Sanderson M.J. (2009). Ion channel regulation of intracellular calcium and airway smooth muscle function. Pulm. Pharmacol. Ther..

[60] Byron K.L., Brueggemann L.I., Kakad P.P., Haick J.M., Wang Y.-X. (2014). Kv7 (KCNQ) Potassium Channels and L-type Calcium Channels in the Regulation of Airway Diameter. Calcium Signaling in Airway Smooth Muscle Cells.

[61] Janssen L.J., Killian K. (2006). Airway smooth muscle as a target of asthma therapy: History and new directions. Respir. Res..

[62] Kume H., Wang Y.-X. (2014). Large-Conductance Calcium-Activated Potassium Channels. Calcium Signaling in Airway Smooth Muscle Cells.

[63] Brueggemann L.I., Cribbs L.L., Schwartz J., Wang M., Kouta A., Byron K.L. (2018). Mechanisms of PKA-dependent potentiation of Kv7.5 channel activity in human airway smooth muscle cells. Int. J. Mol. Sci..

[64] Janssen L.J., Sims S.M. (1992). Acetylcholine activates non-selective cation and chloride conductances in canine and guinea-pig tracheal myocytes. J. Physiol..

[65] Janssen L.J., Sims S.M. (1995). Ca(^2+^)-dependent Cl- current in canine tracheal smooth muscle cells. Am. J. Physiol..

[66] Wang Y.X., Fleischmann B.K., Kotlikoff M.I. (1997). M2 receptor activation of nonselective cation channels in smooth muscle cells: Calcium and Gi/G(o) requirements. Am. J. Physiol.-Cell Physiol..

[67] Kotlikoff M.I., Wang Y.X. (1998). Calcium release and calcium-activated chloride channels in airway smooth muscle cells. Am. J. Respir. Crit. Care Med..

[68] ZhuGe R., Sims S.M., Tuft R.A., Fogarty K.E., Walsh J.V. (1998). Ca21 sparks 12 activate K and Cl channels, resulting in spontaneous transient currents in guinea-pig tracheal myocytes. J. Physiol..

[69] Huang F., Rock J.R., Harfe B.D., Cheng T., Huang X., Jan Y.N., Jan L.Y. (2009). Studies on expression and function of the TMEM16A calcium-activated chloride channel. Proc. Natl. Acad. Sci. USA.

[70] Huang F., Zhang H., Wu M., Yang H., Kudo M., Peters C.J., Woodruff P.G., Solberg O.D., Donne M.L., Huang X. (2012). Calcium-activated chloride channel TMEM16A modulates mucin secretion and airway smooth muscle contraction. Proc. Natl. Acad. Sci. USA.

[71] Wang P., Zhao W., Sun J., Tao T., Chen X., Zheng Y.Y., Zhang C.H., Chen Z., Gao Y.Q., She F. (2018). Inflammatory mediators mediate airway smooth muscle contraction through a G protein-coupled receptor-transmembrane protein 16A-voltage-dependent Ca^2+^ channel axis and contribute to bronchial hyperresponsiveness in asthma. J. Allergy Clin. Immunol..

[72] White T.A., Xue A., Chini E.N., Thompson M., Sieck G.C., Wylam M.E. (2006). Role of transient receptor potential C3 in TNF-alpha-enhanced calcium influx in human airway myocytes. Am. J. Respir. Cell Mol. Biol..

[73] Wang Y.X., Zheng Y.M. (2011). Molecular expression and functional role of canonical transient receptor potential channels in airway smooth muscle cells. Adv. Exp. Med. Biol..

[74] Farley J.M., Miles P.R. (1978). The sources of calcium for acetylcholine-induced contractions of dog tracheal smooth muscle. J. Pharmacol. Exp. Ther..

[75] Dwivedi R., Drumm B.T., Alkawadri T., Martin S.L., Sergeant G.P., Hollywood M.A., Thornbury K.D. (2023). The TMEM16A blockers benzbromarone and MONNA cause intracellular Ca^2+^-release in mouse bronchial smooth muscle cells. Eur. J. Pharmacol..

[76] Shieh C.C., Petrini M.F., Dwyer T.M., Farley J.M. (1992). Cromakalim effects of acetylcholine-induced changes in cytosolic calcium and tension in swine trachealis. J. Pharmacol. Exp. Ther..

[77] Ghosh S., Alkawadri T., McGarvey L.P., Hollywood M.A., Thornbury K.D., Sergeant G.P. (2025). Role of voltage-gated Ca^2+^ channels and Ano1 Ca^2+^-activated Cl-channels in M2 muscarinic receptor-dependent contractions of murine airway smooth muscle. Am. J. Physiol.-Lung Cell Mol. Physiol..

[78] Danielsson J., Perez-Zoghbi J., Bernstein K., Barajas M.B., Zhang Y., Kumar S., Sharma P.K., Gallos G., Emala C.W. (2015). Antagonists of the TMEM16A calcium- activated chloride channel modulate airway smooth muscle tone and intracellular calcium. Anesthesiology.

[79] Danielsson J., Kuforiji A.S., Yocum G.T., Zhang Y., Xu D., Gallos G., Emala C.W. (2020). Agonism of the TMEM16A calcium-activated chloride channel modulates airway smooth muscle tone. Am. J. Physiol.-Lung Cell Mol. Physiol..

[80] Chen Q., Cannell M., van Breemen C. (1992). The superficial buffer barrier in vascular smooth muscle. Can. J. Physiol. Pharmacol..

[81] Chen Q., van Breemen C. (1993). The superficial buffer barrier in venous smooth muscle: Sarcoplasmic reticulum refilling and unloading. Br. J. Pharmacol..

[82] van Breemen C., Chen Q., Laher I. (1995). Superficial buffer barrier function of smooth muscle sarcoplasmic reticulum. Trends Pharmacol. Sci..

[83] Janssen L.J., Betti P.A., Netherton S.J., Walters D.K. (1999). Superficial buffer barrier and preferentially directed release of Ca^2+^ in canine airway smooth muscle. Am. J. Physiol.-Lung Cell. Mol. Physiol..

[84] James P., Inui M., Tada M., Chiesi M., Carafoli E. (1989). Nature and site of phospholamban regulation of the Ca^2+^ pump of sarcoplasmic reticulum. Nature..

[85] Colyer J. (1998). Phosphorylation states of phospholamban. Ann. N. Y. Acad. Sci..

[86] Aickin C.C., Brading A.F. (1982). Measurement of intracellular chloride in guinea-pig vas deferens by ion analysis, 36chloride efflux and micro-electrodes. J. Physiol..

[87] Imaizumi Y., Muraki K., Takeda M., Watanabe M. (1989). Measurement and simulation of non-inactivating Ca current in smooth muscle cells. Am. J. Physiol.-Cell Physiol..

[88] Kume H., Kotlikoff M.I. (1991). Muscarinic inhibition of single KCa channels in smooth muscle cells by a pertussis-sensitive G protein. Am. J. Physiol.-Cell Physiol..

[89] Kume H., Graziano M.P., Kotlikoff M.I. (1992). Stimulatory and inhibitory regulation of calcium-activated potassium channels by guanine nucleotide-binding proteins. Proc. Natl. Acad. Sci. USA.

[90] Zhou X.B., Wulfsen I., Lutz S., Utku E., Sausbier U., Ruth P., Wieland T., Korth M. (2008). M2 muscarinic receptors induce airway smooth muscle activation via a dual, Gbetagamma-mediated inhibition of large conductance Ca^2+^-activated K^+^ channel activity. J. Biol. Chem..

[91] Brueggemann L.I., Kakad P.P., Love R.B., Solway J., Dowell M.L., Cribbs L.L., Byron K.L. (2012). Kv7 potassium channels in airway smooth muscle cells: Signal transduction intermediates and pharmacological targets for bronchodilator therapy. Am. J. Physiol.-Lung Cell. Mol. Physiol..

[92] Brueggemann L.I., Haick J.M., Neuburg S., Tate S., Randhawa D., Cribbs L.L., Byron K.L. (2014). KCNQ (Kv7) potassium channel activators as bronchodilators: Combination with a beta2-adrenergic agonist enhances relaxation of rat airways. Am. J. Physiol.-Lung Cell. Mol. Physiol..

[93] Jones F., Gamper N., Gao H. (2021). Kv7 Channels and Excitability Disorders. Handb. Exp. Pharmacol..

[94] van der Horst J., Greenwood I.A., Jepps T.A. (2020). Cyclic AMP-Dependent Regulation of Kv7 Voltage-Gated Potassium Channels. Front. Physiol..

[95] Song T., Zheng Y.M., Vincent P.A., Cai D., Rosenberg P., Wang Y.X. (2016). Canonical transient receptor potential 3 channels activate NF-κB to mediate allergic airway disease via PKC-α/IκB-α and calcineurin/IκB-β pathways. FASEB J..

[96] Billington C.K., Penn R.B., Hall I.P. (2017). β2 Agonists. Handb. Exp. Pharmacol..

[97] Pera T., Penn R.B. (2014). Crosstalk between beta-2-adrenoceptor and muscarinic acetylcholine receptors in the airway. Curr. Opin. Pharmacol..

[98] Fernandes L.B., Fryer A.D., Hirshman C.A. (1992). M2 muscarinic receptors inhibit isoproterenol-induced relaxation of canine airway smooth muscle. J. Pharmacol. Exp. Ther..

[99] Watson N., Eglen R.M. (1994). Effects of muscarinic M_2_ and M_3_ receptor stimulation and antagonism on responses to isoprenaline of guinea-pig trachea in vitro. Br. J. Pharmacol..

[100] Schramm C.M., Arjona N.C., Grunstein M.M. (1995). Role of muscarinic M2 receptors in regulating beta-adrenergic responsiveness in maturing rabbit airway smooth muscle. Am. J. Physiol.-Lung Cell. Mol. Physiol..

[101] Sarria B., Naline E., Zhang Y., Cortijo J., Molimard M., Moreau J., Therond P., Advenier C., Morcillo E.J. (2002). Muscarinic M2 receptors in acetylcholine-isoproterenol functional antagonism in human isolated bronchus. Am. J. Physiol.-Lung Cell. Mol. Physiol..

[102] Brown S.M., Koarai A., Sturton R.G., Nicholson A.G., Barnes P.J., Donnelly L.E. (2013). A role for M(2) and M(3) muscarinic receptors in the contraction of rat and human small airways. Eur. J. Pharmacol..

[103] Torphy T.J., Rinard G.A., Rietow M.G., Mayer S.E. (1983). Functional antagonism in canine tracheal smooth muscle: Inhibition by methacholine of the mechanical and biochemical responses to isoproterenol. J. Pharmacol. Exp. Ther..

[104] Torphy T.J., Zheng C., Peterson S.M., Fiscus R.R., Rinard G.A., Mayer S.E. (1985). Inhibitory effect of methacholine on drug-induced relaxation, cyclic AMP accumulation, and cyclic AMP-dependent protein kinase activation in canine tracheal smooth muscle. J. Pharmacol. Exp. Ther..

[105] Panettieri R.A. (2015). Bronchodilators, receptors and cross-talk: Together is better?. Postgrad. Med..

[106] Alkawadri T., Wong P.Y., Fong Z., Lundy F.T., McGarvey L.P., Hollywood M.A., Thornbury K.D., Sergeant G.P. (2022). M2 Muscarinic Receptor-Dependent Contractions of Airway Smooth Muscle are Inhibited by Activation of β-Adrenoceptors. Function.

[107] Whicker S.D., Compton M.R., Seale J.P., Black J.L. (1990). Effect of sensitization and aerosol antigen challenge in guinea-pigs: Studies of airway receptor function and characteristics. Pulm. Pharmacol..

[108] Haddad E.-B., Mak J.C.W., Belvisi M.G., Nishikawa M., Rousell J., Barnes P.J. (1996). Muscarinic and adrenergic receptor expression in peripheral lung from normal and asthmatic patients. Am. J. Physiol.-Lung Cell. Mol. Physiol..

[109] Donfack J., Kogut P., Forsythe S., Solway J., Ober C. (2003). Sequence variation in the promoter region of the cholinergic receptor muscarinic 3 gene and asthma and atopy. J. Allergy Clin. Immunol..

[110] Hakonarson H., Herrick D.J., Grunstein M.M. (1995). Mechanism of impaired beta-adrenoceptor responsiveness in atopic sensitized airway smooth muscle. Am. J. Physiol.-Lung Cell. Mol. Physiol..

[111] Chiba Y., Sakai H., Misawa M. (2001). Possible involvement of G(i3) protein in augmented contraction of bronchial smooth muscle from antigen-induced airway hyperresponsive rats. Biochem. Pharmacol..

[112] Barnes P.J., Chung K.F., Page C.P. (1988). Inflammatory mediators and asthma. Pharmacol. Rev..

[113] Broide D.H., Lotz M., Cuomo A.J., Coburn D.A., Federman E.C., Wasserman S.I. (1992). Cytokines in symptomatic asthma airways. J. Allergy Clin. Immunol..

[114] Kips J.C., Tavernier J., Pauwels R.A. (1992). Tumor necrosis factor (TNF) causes bronchial hyperresponsiveness in rats. Am. Rev. Respir. Dis..

[115] Borish L., Joseph B.Z. (1992). Inflammation and the allergic response. Med. Clin. North Am..

[116] Hamblin A.S. (1991). The role of cytokines in asthma. Ann. N. Y. Acad. Sci..

[117] Hakonarson H., Herrick D.J., Serrano P.G., Grunstein M.M. (1996). Mechanism of cytokine-induced modulation of beta-adrenoceptor responsiveness in airway smooth muscle. J. Clin. Investig..

[118] Huang D., Zhang L., Liu Y., Wang J., Zhang J., Baines K.J., Liu G., Hsu A.C.-Y., Wang F., Chen Z. (2024). Activated non-neuronal cholinergic system correlates with non-type 2 inflammation and exacerbations in severe asthma. Ann. Allergy Asthma Immunol..

[119] Spina D. (2014). Current and novel bronchodilators in respiratory disease. Curr. Opin. Pulm. Med..

